# Welcome to the *Journal of Personalized Medicine*: A New Open-Access Platform for Research on Optimal Individual Healthcare

**DOI:** 10.3390/jpm1010001

**Published:** 2011-03-28

**Authors:** Urs A. Meyer

**Affiliations:** *Founding Editor-in-Chief of Journal of Personalized Medicine*, Pharmacology/Neurobiology, Biozentrum, University of Basel, Klingelbergstrasse 50/70, CH-4056 Basel, Switzerland; E-Mail: urs-a.meyer@unibas.ch; Tel.: +41-61-267-2220; Fax: +41-61-267-2208

A new vision of personalized medicine or personalized healthcare has evolved as a consequence of remarkable recent advances in technologies that allow to look at individual variation across the entire human genome and to identify personal risk factors behind many diseases and responses to therapy. These advances have greatly increased our understanding of how interactions between the entire genome and nongenomic factors result in health and disease and in therapeutic response. The challenge is now to translate this knowledge into benefits for the individual patient. I expect the *Journal of Personalized Medicine* to become the premier venue for the rapid and freely accessible publication of high quality manuscripts dealing with this vision for scientists around the world.

Personalized medicine is not a new idea or revolution as physicians have always treated patients on the basis of the available knowledge and the probability that a certain medication will benefit the patient. The historical writings of Hippocrates, Garrod, Osler and others already emphasized the centrality of “treating the patient, not the disease”. What we have witnessed in the last decade, however, is a breathtaking acceleration in understanding human genetic diversity as the result of a technological revolution.

Densely packed microarrays with up to five million oligonucleotides are able to detect millions of sequence variants such as single nucleotide polymorphisms (SNPs) or copy number variants (CNVs), other sequence changes or RNAs for the price of a “normal” laboratory test. This allows genome wide association studies (GWAS) or gene expression studies in thousands of patients. So-called next generation sequencing technologies have reduced the cost of reading DNA literally a million-fold (!) since the end of the Human Genome Project. Genome sequences by the thousands are thus on the horizon. Approximately 3000 genomes were sequenced by the end of 2010 and it is predicted that by the end of 2011, 30000 human genome sequences will be available in public databases [1]. The $1000 human genome sequence is most likely possible within the next three years.

The information from GWAS and sequencing allows more precise haplotype maps for different populations to interpret genotyping results. Another consequence of affordable sequencing is the Cancer Genome Atlas (TCGA, http://cancergenome.nih.gov) [2] and the International Cancer Genome Consortium (ICGC, http://icgc.org) [3] with the goal to characterize genomic changes across a range of different cancer types. Just about one year ago, the first clinical assessment of a patient incorporating a personal genome sequence was published [4]. Is this the path for the future of medicine? A more controversial spin-off of low-cost genotyping and sequencing is what we call “consumer genomics” or DTC (direct to consumer) genomics. Virtually dozens of companies offer fee-for-service genotyping to whoever sends in a saliva sample for as low as $200. The customer receives an interpretation of his statistical personal genetic risk for several dozens of diseases and possible pharmacogenetic problems. There are obviously considerable questions and disputes regarding this unregulated business of selling questionable, and for the patient mostly useless, information.

What have we learned so far from the wave of new sequence data? First of all, there is no “normal” human genome sequence; we are all “mutants”. The similarity at the sequence level of two individuals is approximately 1%, or 60 million base pairs for a diploid genome. The GWAS and sequencing studies have identified hundreds of gene variants which contribute to disease. In many cases, but not consistently, this has resulted in a better understanding of the involved pathways and their pathogenic role. Molecular diagnostic tests or biomarkers for more precise diagnosis or for therapeutic decisions are the tangible results from these discoveries. But what we also have learned from the flurry of GWAS and sequence data in health and disease is that common genetic variation (e.g., SNPs occurring in the population at 5% frequency) have only a limited role in determining the genetic predisposition to common diseases such as type 2 diabetes, essential hypertension, *etc.* On the other hand, gene variants that are very rare in the general population can have outsized effects on the predisposition to certain other diseases, examples are schizophrenia and autism.

How are we going to use this knowledge to improve human health? The task is indeed enormous. For many common diseases (cancer, diabetes, cardiovascular disease, rheumatoid arthritis, dementia, *etc.*) there are no effective or curing treatments. Even though there is a dearth of new drugs, the pharmaceutical industry is unable to bring more innovative new drugs to the market. There has been a continuous decline of new drug approvals by the Food and Drug Administration (FDA) from 56 in 1996 to just 21 last year, including both NMEs (new molecular entities) and biologics, in spite of ever higher investments in research and development [5]. The present model of drug research and development is not working and profound changes are needed. Personalized medicine, with its goal to predict responders and non-responders to therapy and to develop biomarkers that can be used as guides in the process of drug development, is one strategy to pursue.

There is no consensus definition of personalized medicine. My “personal” view is to see personalized medicine very broadly as a comprehensive, prospective approach in order to prevent, diagnose and treat disease to achieve an optimal result for the individual. It starts with the assessment of the individual disease risk to allow early diagnosis and/or preventive measures. In the field of early diagnosis there has been a very recent technological breakthrough, namely the sequencing of fetal genomes with DNA from the mother's blood. Cell-free fetal DNA (ccffDNA) occurs as 5-10% of total free DNA in the blood of the mother already early in pregnancy. It allows the diagnosis of trisomy 21 and other aneuploidies [6], can test for Rh factor of the fetus and could be used for early identification of cystic fibrosis and β-thalassemia, diseases which can be treated *in utero*. Evidently, non-invasive prenatal genetic diagnosis has arrived.

The second tenet of personalized medicine is to increase diagnostic precision by defining subphenotypes of the disease with prognostic and therapeutic implications, for instance by testing tumor biopsies for gene signatures (OncotypeDX, Mammaprint) which give information on the likelihood of chemotherapy benefit and recurrence risk in breast cancer. The validity of this approach has been well documented.

A third and often considered the principal aspect of personalized medicine is the tailoring of treatment to the individual characteristics of each patient. This is of course much dependent on the ability to classify patients into subpopulations with predictable response to a specific treatment. The field of pharmacogenetics/pharmacogenomics has made major contributions to this problem for more than 50 years [7]. This allows treatment of only the likely responders, to avoid adverse reactions and expensive treatments in non-responders. This approach has considerable implications in how prospective clinical trials will be designed in the future. Both diagnosis and therapy have to take into account not only the potential variation in pharmacokinetics but also the presence as well as structural and functional variation of the drug target (e.g. epidermal growth factor receptor in cancer treatments). Moreover, the influence of host factors (age, sex, body mass index, previous diseases, *etc.*) and environmental factors (e.g. smoking, alcohol, nutrition, concomitant drugs, *etc.*) have to be considered. For instance, the prediction of the individual dose of warfarin uses multiple algorithms that include genotypes for four genes (CYP2C9, VKORC1, CYP4F2, GGCX), six host factors and six clinical or environmental factors (www.warfarindosing.org) [8]. Combined, these parameters can predict approximately 50 to 60% of the individual dose, an important improvement over the trial and error approach.

The fourth point in the concept of personalized medicine is the proper evaluation of objective and subjective clinical outcomes and ultimately the clinical utility or practicality of individualized health care.

Personalized medicine will have reached its goals if one day personalized medicine will be simply just “medicine”.

I have accepted to be the founding Editor-in-Chief of the *Journal of Personalized Medicine* because of my long-time involvement in optimizing therapeutic decisions as a clinical pharmacologist and in my research in pharmacogenetic diseases and pharmacogenomics. My laboratory developed a first pharmacogenetic DNA test in 1990 [9] and I have walked the long road from association to a clinically useful molecular diagnostic test many times. I am aware of the immense potential but also of the many questions and problems of the recent advances in personalized medicine. Some advances will be uncontroversial, such as the individualization of drug choice and dose. Others will be important for drug development, for instance the identification of genetic risk factors for now poorly treatable diseases that allow the definition of new targets for therapy. I am less certain of how we will handle the ethical implications when millions of people have their genomes sequenced and what it means to those who are confronted with serious genetic risks, which are just risks but not certainties. I foresee that we will discuss all these issues in the *Journal of Personalized Medicine*.

In launching this open access journal, the members of the Editorial Board and I are hopeful that we can attract high quality manuscripts with the most novel data. We offer an efficient peer-review and editorial decision and to make these publications rapidly and freely available to anyone anywhere in the world. In addition, publication fees for the first two volumes will be waived. We hope that you will join us in making this journal a success.
